# Rhinosclerome du cavum avec expression ganglionnaire cervicale: à propos d’un cas

**DOI:** 10.11604/pamj.2018.30.116.13108

**Published:** 2018-06-12

**Authors:** Omar Lassikri, Jalila Benayad, Omar Lachhab, Ali El Ayoubi, Mohamed Anas Benbouzid, Leila Essakalli

**Affiliations:** 1 Service d’Oto-Rhino-Laryngologie et Chirurgie Cervico-Faciale, CHU Ibn Sina, Rabat, Maroc

**Keywords:** Rhinosclérome, cavum, adénopathie cervicale, traitement, Rhinoscleroma, cavum, cervical lymphadenopathy, treatment

## Abstract

Le rhinosclérome est une affection granulomateuse spécifique et chronique d'évolution insidieuse, dont l'agent pathogène est le Klebsiella rhinoscleromatis. Il touche avec prédilection les fosses nasales, et pose parfois un problème de diagnostic positif. Nous rapportons le cas d'une patiente de 19 ans, qui présente un rhinosclérome atypique par sa localisation nasopharyngée rare, et son association exceptionnelle à une adénopathie cervicale sous angulo-mandibulaire droite. La mise en évidence des cellules de MIKULICZ à l'examen anatomo-pathologique a permis de poser le diagnostic. La patiente a été mise sous traitement antibiotique à base de ciprofloxacine pendant 16 semaines, associée à un nettoyage des fosses nasales par sérum physiologique. L'évolution était favorable avec un recul de 14 mois.

## Abstract (en)

Rhinoscleroma is a specific granulomatous and chronic disorder with insidious evolution. It is causes by pathogen Klebsiella rhinoscleromatis. It mainly occurs in the nasal cavities and positive diagnosis is sometimes problematic. We report the case of a 19 year old female patient presenting with rhinoscleroma considered atypical due to its rare nasopharyngeal localization and its exceptional association with cervical lymphadenopathy in the right submandibular angle region. Anatomopathological exam revealed Mikulicz's cells, thus enabling the diagnosis. The patient underwent antibiotic therapy with ciprofloxacin for 16 weeks associated with washing of nasal cavities with physiological saline solution. Patient's outcome was favorable during the 14-month follow-up period.

## Introduction

Le rhinosclérome est une affection granulomateuse rare et chronique évoluant de façon insidieuse et lente. Elle se développe au niveau des fosses nasales dans 95% des cas [1], mais elle peut se localiser dans d'autre site du tractus respiratoire. Son agent pathogène est le Klebsiella rhinoscleromatis, qui est une coccobacille Gram négatif ayant un tropisme pour les voies aériennes supérieures. C'est une affection spécifique caractérisée cliniquement par un granulome à évolution pseudo-tumorale, et histologiquement par la présence des cellules de MIKULICZ. Elle pose parfois un problème de diagnostic positif dont la confirmation ne peut être qu'histologique. Son traitement est essentiellement médical.

## Patient et observation

**Figure 1:**
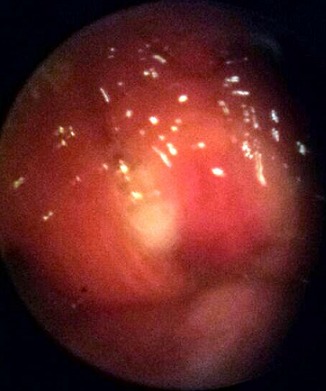
Aspect endoscopique du processus bourgeonnant, comblant le cavum avec issu de secrétions purulentes

**Figure 2:**
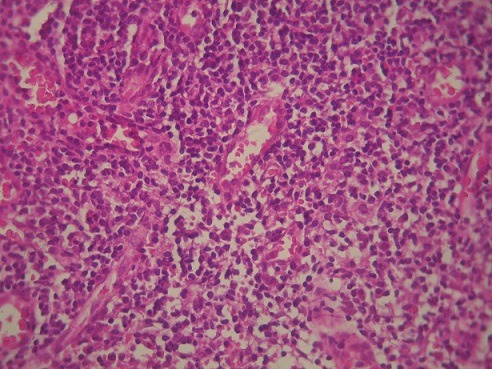
Image anatomopathologique montrant le processus lymphoplasmocytaire avec des cellules de Mikulicz

Patiente de 19 ans, immunocompétente, qui consulte pour une obstruction nasale bilatérale d'installation progressive avec rhinorrhée séro-muqueuse, associée à une masse cervicale sous angulo-mandibulaire droite évoluant depuis 8 mois. La rhinoscopie antérieure a objectivé une rhinorrhée claire. L'endoscopie nasale a mis en évidence un processus polypoïde bourgeonnant et infecté, comblant le cavum et les choanes (Figure 1). L'échographie cervicale a objectivé de multiples adénopathies cervicales droites jugulo-carotidiennes, sous parotidienne, et sous angulo-mandibulaire qui est la plus grande mesurant 22x14mm. Une biopsie du processus nasopharyngé sous anesthésie locale a été réalisée, et l'examen anatomopathologique a révélé la présence des plages lymphoplasmocytaires diffuses parsemées de nombreux histiocytes à cytoplasme volumineux vacuolaires, renfermant des structures bacillaires correspondant à des cellules de MIKULICZ pathognomoniques du rhinosclérome (Figure 2). Le bilan biologique (numération de la formule sanguine, ionogramme sanguin, CRP) était normal. La patiente a été mise sous antibiothérapie à base de ciprofloxacine 500 mg x 2 par jour pendant 16 semaines. Avec un recul de 14 mois sous contrôle par endoscopies nasales et échographies cervicales, l'évolution était favorable marquée par la disparition complète de la masse nasopharyngée au 4éme mois et des adénopathies cervicales au 8éme mois. Aucun cas similaire n´a été décelé à l'enquête familiale.

## Discussion

Malgré qu'il soit de plus en plus rare, le rhinosclerome sévit encore à l'état endémique dans l'Amérique centrale, l'Amérique du Sud, l'Europe de l'Est, l'Afrique centrale, le Maghreb, et le Moyen-Orient [2-4]. Néanmoins la croissance des mouvements de population a donné à cette granulomatose un caractère cosmopolite. Les communautés de bas niveau socio-économique sont les plus touchés en raison du manque d'hygiène, des déficits nutritionnels et immunitaires, qui sont les principaux facteurs favorisants [3]. Le mode d'infection n'est pas bien défini, mais l'atteinte de plusieurs membres de la même famille, pourrait soutenir l'hypothèse d'une transmission par un contact direct étroit et prolongé [1,5]. Le Rhinosclérome touche essentiellement les adultes jeunes dans les troisième et quatrième décennies de la vie (85% des cas) [5], avec une prédominance féminine de 60% des cas [2, 6]. La fréquence des cas familiaux est estimée à 20% [7]. L'évolution clinique est lente et insidieuse expliquant le retard du diagnostic souvent fait au stade de lésions pseudo-tumorales, comme c'est le cas de notre patiente. L'atteinte nasale est la plus fréquente estimée à 95% des cas [1]. Les formes naso-pahryngées sont rares, ainsi dans notre contexte épidémiologique nous avons évoqué le diagnostic du cancer du cavum et la tuberculose. Les localisations laryngées ou trachéobronchiques sont également rares mais graves [2]. Contrairement aux autres granulomatoses, le Rhinosclerome n´affecte pas le système lymphatique. Le dépôt de tissu fibreux autour des infiltrations granulomateuses bloque les lymphatiques, empêchant l'atteinte des ganglions par les bacilles [8]. De nombreux aspects de la maladie sont encore en suspens, et c'est le cas de notre patiente dont l'atteinte ganglionnaire était présente. Les signes cliniques et endoscopiques étant non spécifiques, le diagnostic est anatomopathologique par la mise en évidence des cellules spécifiques de MIKULICZ [6], qui sont des cellules spumeuses au sein du cytoplasme des plasmocytes avec des inclusions acidophiles très réfringentes réalisant des aspects pathognomoniques dits corps de RUSSEL. La recherche et le typage de klebsiella rhinoscleromatis au niveau des sécrétions nasales ne sont positifs que dans 50 à 60% des cas [4]. Le diagnostic immunologique utilise la réaction de fixation du complément qui est positive dans 92% des cas. L'intérêt de la TDM, est d'apporter des renseignements sur la topographie et l'extension des lésions. Le bilan biologique permet de rechercher une anémie hypochrome hyposidérémique, une lymphocytose ou une hyperéosinophilie qui sont souvent associées [9]. La prise en charge thérapeutique du rhinosclérome est essentiellement médical, basée sur une antibiothérapie par voie générale prolongée pendant plusieurs mois [2, 3], associée à un nettoyage abondant des fosses nasales au sérum physiologique. La rifampicine et les fluoroquinolones restent à l'heure actuelle les meilleurs traitements en raison de leur concentration élevée au niveau des macrophages. La durée du traitement n'est pas encore bien codifiée, elle varie selon les auteurs entre 6 semaines et 6 mois. En général la stérilisation du foyer infectieux est obtenue à partir du 3^ème^ mois [9]. D'autres antibiotiques sont également efficaces, mais du fait de leur toxicité cochléo-véstibulaire ou hématologique ils sont moins utilisés: La streptomycine, cotrimoxazole, oxytétracycline, sulfamides [9]. Un traitement martial est parfois associé en cas d'anémie. La corticothérapie peut être utiliser pour minimiser le processus de sclérose. La tendance à la récidive est la règle, qui peut se produire dans 41% des cas entre 1-3 ans [5]. Après un recul de 14 mois chez notre patiente, aucune récidive locale n'a été décelé à 10 mois de l'arrêt du traitement. Le traitement chirurgical s'adresse aux lésions fibroscléreuses dont le but est la perméabilisation et le calibrage des fosses nasales [10].

## Conclusion

Le rhinosclérome est une affection bénigne qui paraît de plus en plus rare. Sa localisation rhinopharyngée avec atteinte ganglionnaire est inhabituelle, pouvant poser un problème de diagnostic positif, d'où l'intérêt d'y penser systématiquement devant toute tumeur du cavum. Le traitement médical précoce et adapté, permet d'améliorer le pronostic et d'éviter l'évolution vers des séquelles invalidantes.

## Conflits d’intérêts

Les auteurs ne déclarent aucun conflit d'intérêts.
